# Transcriptomic data of BT549 triple negative breast cancer cells treated with 20 µM NU7441, a DNA-dependent kinase inhibitor

**DOI:** 10.1016/j.dib.2024.110183

**Published:** 2024-02-13

**Authors:** Kunyan Li, Shuailong Zhang, Yupeng Gu, Jinyan Wang, Yanqin Yang, Weifeng Mao

**Affiliations:** a College of Basic Medical Sciences, Dalian Medical University, Dalian 116044, PR China; b Department of Oncology, the Second Associated Hospital, Dalian Medical University, Dalian, 116011, PR China

**Keywords:** DNA-PK, Breast cancer, RNA-seq, NU7441

## Abstract

DNA-dependent protein kinase catalytic subunit (DNA-PK) is a multifunctional serine‑threonine protein kinase that plays roles in non-homologous end joining of DNA repair in cells. NU7441 is a specific DNA-PKcs inhibitor. We investigated the effects of NU7441 on the transcriptome of BT549 triple negative breast cancer cells. Total RNA extracted from NU7441-treated or control BT549 cells was processed for preparation of sequencing libraries. Assessment of read quality was performed using fastqc tool. Trimming and filtering low-quality reads were performed using fastp. Reads were aligned by hisat2. SAM files were converted to BAM files using Samtools. The gene differential expression analysis, Gene Ontology (GO) analysis and KEGG pathway analysis were performed. After NU7441 treatment, total number of 2045 differential genes were selected according to |log2(FoldChange)| >= 1 & padj<= 0.05, among which 1365 genes were down-regulated and 680 genes were up-regulated. The differential expression genes in pattern recognition receptors (PRRs) immune responses signals, including NOD-like receptor signaling, Toll-like receptor signaling, RIG-I-like receptor signaling and cytosolic DNA-sensing pathways were noted in this paper.

Specifications Table

**Table:** 

Subject	Biological sciences /Cancer Research
Specific subject area	Transcriptome and global gene expression of BT549 cells treated with NU7441 and control.
Data format	Raw sequence (FASTQ), Raw gene count matrix
Type of data	Table, text file, figure
Data collection	Total RNA extracted from NU7441 or vehicle-treated cells was processed for preparation of sequencing libraries using TruSeqTM RNA sample preparation Kit from Illumina (San Diego, USA). Assessment of read quality was performed using fastqc tool. Trimming and filtering low-quality reads were performed using fastp. Reads were aligned by hisat2. SAM files were converted to BAM files using Samtools. The RNA-seq raw data are available from NCBI (accession number in NCBI is PRJNA872854).
Data source location	• Institution:College of Basic Medical Sciences, Dalian Medical University • City/Town/Region:Dalian • Country:China Latitude and longitude (and GPS coordinates, if possible) for collected samples/data: 121.314834,−38.804539 • (38°48′16.340″ N; 121°18′53.402″ E)
Data accessibility	Raw data is within paper and Medeley Data. Repository name: Medeley Data Data identification number: doi:10.17632/xjz27scmjd.1 Direct URL to data: https://data.mendeley.com/datasets/xjz27scmjd/1
Related research article	Chao Ma, Yuanhua Qin, Ying Wang, Chuanliang Zhu, Xingjie Gao, Pingping Zhang, Yupeng Gu, Shuailong Zhang, Jintao Lin, Jiahui Wang, Weifeng Mao, Targeting DNA-PKcs promotes anti-tumoral immunity via triggering cytosolic DNA sensing and inducing an inflamed tumor immuno-microenvironment in metastatic triple negative breast cancer,Genes & Diseases, ISSN 2352-3042, September 2023. https://doi.org/10.1016/j.gendis.2023.01.001[1].

## 1 Value of the Data


 

• This information uncovers a range of downstream analyses, including annotation, differential expression, pathway investigations between BT549 breast cancer cells treated with NU7441 and control. These data are valuable for the understanding the effects of inhibition of DNA-PK on the global gene expressions and pathways.
• This dataset reported the differential genes in pattern recognition receptors (PRRs) immune responses signals, including NOD-like receptor signaling, Toll-like receptor signaling, RIG-I-like receptor signaling and cytosolic DNA-sensing pathways in BT549 cells treated with NU7441 and control. The relationship of DNA damage response and PRRs immune signals were not well studied. As DNA-PK is a vital sensor in repair of DNA double strand breaks, this dataset allow in-depth exploration of the relationship between DNA damage response and cytosolic nucleic acids-sensing immune signals.
• DNA-PK is a vital factor in non-homologous end joining (NHEJ) repair, these dataset would be valuable to the investigation of NHEJ repair and the generation of cytosolic DNA, well as the relationship of DNA damage repair and the innate immunity stimulated by cytosolic DNA.
• This transcriptome sequences will function as essential references and valuable reservoirs for the investigation of the inhibitions of DNA-PK in innate immunity, tumoral immune-microenvironment and breast cancer immunotherapy.

## 2 Background

In the published original research article, the DNA-PK inhibitor, NU7441, promoted the inflammation of breast cancer microenvironment. The primary objective of this study is to analyse the effects of DNA-PK inhibitor, NU7441, on the global gene expressions in breast cancer cells and identified the cellular functions and pathways regulated by NU7441 in breast cancer cells. To achieve this goal, the genes of differential expressions were selected and KEGG and GO analysis were performed. The genes in pattern recognition receptors (PRRs) immune responses signals, including NOD-like receptor signaling, Toll-like receptor signaling, RIG-I-like receptor signaling and cytosolic DNA-sensing pathways were noted. This study aims to identify differentially expressed genes and elucidate the distinct immune responses signals and genes triggered by NU7441.

## 3 Data Description

**Fig. 1:**
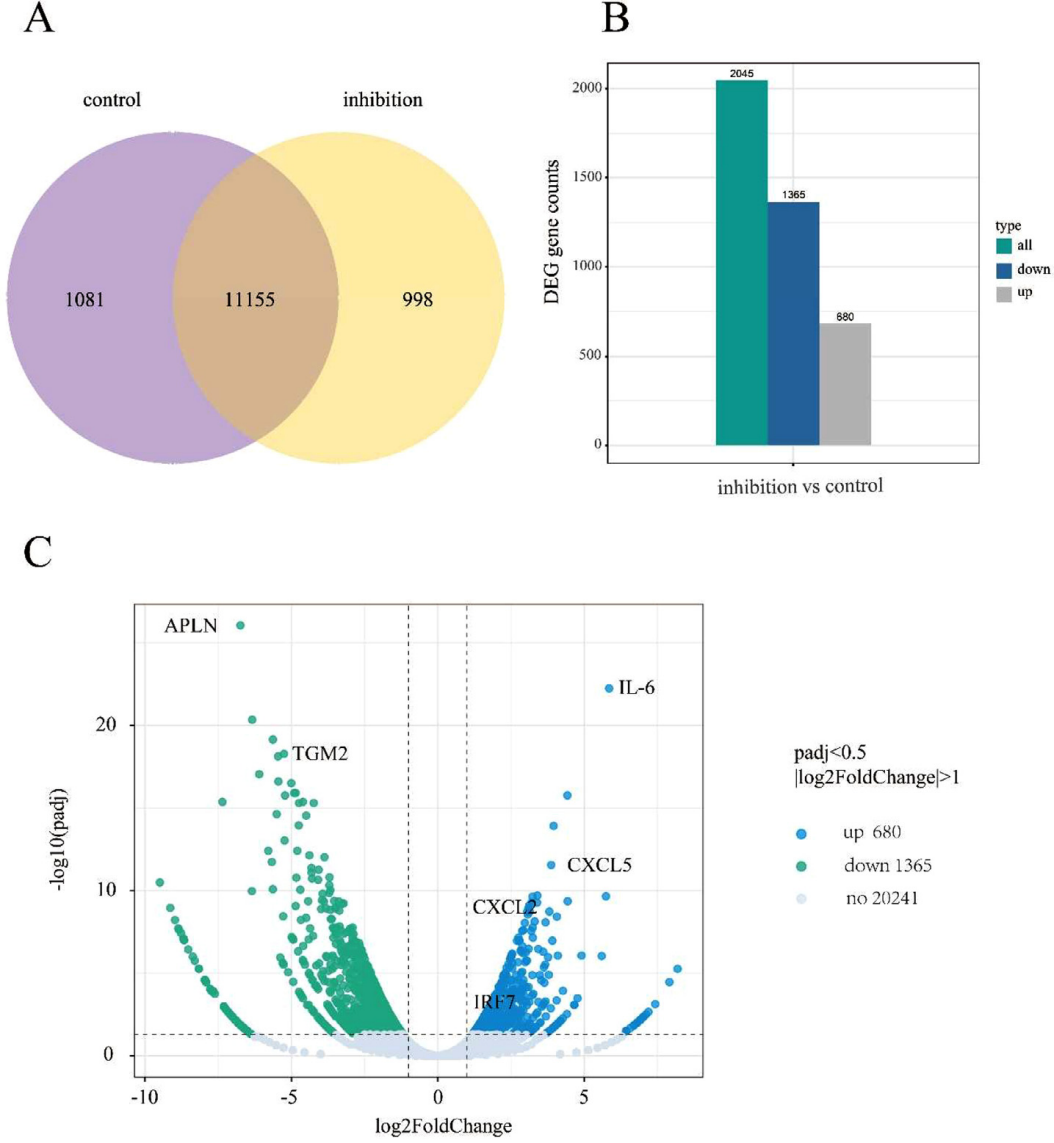
(A) Venn plots of gene co-expression in NU7441-added and control cells, the overlapping area shows the number of co-expressed genes in the two samples. Control represents BT549 cells without drug treatment and inhibition represents BT549 cells after NU7441 treatment (B). Histograms of genes differentially expressed in the NU7441-treated group compared to the control group. The differential genes selected according to |log2(FoldChange)| >= 1 & padj<= 0.05.(C)Volcano plot of differential genes in the NU7441-treated group compared to the control group. 680 genes were up-regulated and 1365 genes were down-regulated in BT549 cells treated with NU7441 according to |log2(FoldChange)| >= 1 & padj<= 0.05.

This dataset consists of the differential gene expressions in BT549 cells treated with NU7441 and control (Fig 1A). The total number of differential genes were selected according to |log2(FoldChange)| >= 1 & padj<= 0.05, and there were 680 genes up-regulated and 1365 genes down-regulated in BT549 cells treated with NU7441 (Fig 1B,C). The gene ontology analysis based on molecular functions, biological processes and cellular components were performed (Table 1, Table 2, Table 3). The genes of differential expressions in NOD-like receptor signaling pathway were noted in Table 4. The genes of differential expressions in Toll-like receptor signalling pathway were noted in Table 5. The genes of differential expressions in RIG-I-like signalling pathway were noted in Table 6. The genes of differential expressions in cytosolic DNA signalling pathway were noted in Table 7. The RNA-seq raw data were deposited and available from NCBI database (accession number PRJNA872854).

**Table 1:** Gene ontology analysis based on molecular functions.

Gene set name	Number of genes in the gene set	Pvalue
alpha-beta T cell activation	26	1.29E-07
inflammatory response	88	2.62E-07
regulation of leukocyte migration	33	6.64E-07
regulation of leukocyte activation	69	2.37E-06
T cell activation	62	1.08E-05
regulation of alpha-beta T cell activation	17	1.23E-05
positive regulation of T cell activation	33	4.60E-05
positive regulation of leukocyte migration	23	7.14E-05
CD4-positive, alpha-beta T cell activation	15	0.000117533
regulation of chemotaxis	32	0.000129057
regulation of leukocyte chemotaxis	20	0.000167479
positive regulation of leukocyte activation	42	0.000264027
regulation of CD4-positive, alpha-beta T cell activation	10	0.000625464
positive regulation of inflammatory response	16	0.002067006
positive regulation of neutrophil migration	8	0.003458071

**Table 2:** Gene ontology analysis based on biological process.

Gene set name	Number of genes in the gene set	Pvalue
extracellular matrix	83	3.71E-12
nucleosome	21	6.21E-05
cell surface	103	4.12E-10
condensed chromosome, centromeric region	35	6.31E-08
cell division site	19	1.56E-06
endoplasmic reticulum lumen	47	2.82E-06
external side of plasma membrane	36	5.74E-06
cortical cytoskeleton	22	1.31E-05
condensed chromosome	47	3.00E-05
kinetochore	34	4.37E-05
collagen trimer	16	6.20E-05
chromosome, centromeric region	43	0.000677028
extracellular matrix component	26	0.000156661
chromosomal region	63	0.000288109
side of membrane	60	0.000372487

**Table 3:** Gene ontology analysis based on cellular component.

Gene set name	Number of genes in the gene set	Pvalue
glycosaminoglycan binding	35	2.42E-06
receptor regulator activity	57	2.65E-06
growth factor binding	30	4.32E-06
receptor ligand activity	53	4.71E-06
transmembrane signaling receptor activity	87	9.48E-06
heparin binding	28	1.38E-05
insulin-like growth factor binding	11	5.79E-05
actin binding	62	7.13E-05
insulin-like growth factor I binding	7	0.000150752
organic acid binding	31	0.000176045
voltage-gated potassium channel activity	15	0.0002263
carboxylic acid binding	30	0.000354185
collagen binding	15	0.000513855
G-protein coupled receptor activity	42	0.000592144
sulfur compound binding	35	0.000606911

**Table 4:** List of genes enriched in NOD-like receptor signaling pathway.

Gene symbol	Description of the gene	Log2 (ratio)	Pvalue
IL6	interleukin 6	5.86	5.09E-27
CXCL2	C-X-C motif chemokine ligand 2	3.11	3.50E-12
CXCL3	C-X-C motif chemokine ligand 3	3.14	5.94E-12
NAMPT	nicotinamide phosphoribosyltransferase	2.52	6.44E-09
NFKBIA	NFKB inhibitor alpha	2.25	1.99E-07
CXCL8	C-X-C motif chemokine ligand 8	1.96	6.04E-06
ITPR1	inositol 1,4,5-trisphosphate receptor type 1	1.93	6.10E-06
IRF7	interferon regulatory factor 7	1.89	2.34E-05
ANTXR1	ANTXR cell adhesion molecule 1	1.77	2.90E-05
TNFAIP3	TNF alpha induced protein 3	1.50	0.000516082
CXCL1	C-X-C motif chemokine ligand 1	1.34	0.001341218
GABARAPL1	GABA type A receptor associated protein like 1	1.38	0.001378212
TRPM7	transient receptor potential cation channel subfamily M member 7	1.30	0.002128185
BIRC3	baculoviral IAP repeat containing 3	−3.32	1.62E-09
IKBKE	inhibitor of nuclear factor kappa B kinase subunit epsilon	−2.85	4.23E-08
TXNIP	thioredoxin interacting protein	−2.19	8.36E-07
OAS3	2′−5′-oligoadenylate synthetase 3	−2.83	1.74E-06
ANTXR2	ANTXR cell adhesion molecule 2	−1.98	4.42E-06
MYD88	MYD88 innate immune signal transduction adaptor	−1.80	6.16E-05
ITPR3	inositol 1,4,5-trisphosphate receptor type 3	−2.29	0.000192772
HSP90AA1	heat shock protein 90 alpha family class A member 1	−1.46	0.000498703
TRAF5	TNF receptor associated factor 5	−1.78	0.000556287
NLRX1	NLR family member X1	−1.5	0.001796524
TMEM173	transmembrane protein 173	−2.63	0.002370371
TRIP6	thyroid hormone receptor interactor 6	−1.21	0.003717912

**Table 5:** List of genes enriched in Toll-like receptor signaling pathway.

Gene symbol	Description of the gene	Log2 (ratio)	pvalue
IL6	interleukin 6	5.86	5.09E-27
NFKBIA	NFKB inhibitor alpha	2.25	1.99E-07
CXCL8	C-X-C motif chemokine ligand 8	1.96	6.04E-06
IRF7	interferon regulatory factor 7	1.89	2.34E-05
MAP3K8	mitogen-activated protein kinase 8	1.83	6.12E-05
FOS	Fos proto-oncogene, AP-1 transcription factor subunit	1.80	7.95E-05
PIK3R1	phosphoinositide-3-kinase regulatory subunit 1	1.30	0.001935018
MYD88	MYD88 innate immune signal transduction adaptor	−1.80	6.16E-05
IKBKE	inhibitor of nuclear factor kappa B kinase subunit epsilon	−2.85	4.23E-08
MAP2K3	mitogen-activated protein kinase 3	−1.57	0.000198172
MAP2K6	mitogen-activated protein kinase 6	−2.03	0.001726885
IL12A	interleukin 12A	−2.90	0.003515158

**Table 6:** List of genes enriched in RIG-I-like receptor signaling pathway.

Gene symbol	Description of the gene	Log2 (ratio)	pvalue
NFKBIA	NFKB inhibitor alpha	2.25	1.99E-07
CXCL8	C-X-C motif chemokine ligand 8	1.96	6.04E-06
IRF7	interferon regulatory factor 7	1.89	2.34E-05
IKBKE	inhibitor of nuclear factor kappa B kinase subunit epsilon	−2.85	4.23E-08
RNF125	ring finger protein 125	−3.16	5.36E-05
ISG15	ISG15 ubiquitin like modifier	−1.37	0.001460095
NLRX1	NLR family member X1	−1.50	0.001796524
TMEM173	transmembrane protein 173	−2.63	0.002370371
IL12A	interleukin 12A	−2.90	0.003515158

**Table 7:** List of genes enriched in Cytosolic DNA-sensing pathway.

Gene symbol	Description of the gene	Log2 (ratio)	pvalue
IL6	interleukin 6	5.86	5.09E-27
NFKBIA	NFKB inhibitor alpha	2.25	1.99E-07
IRF7	interferon regulatory factor 7	1.89	2.34E-05
IKBKE	inhibitor of nuclear factor kappa B kinase subunit epsilon	−2.85	4.23E-08
TMEM173	transmembrane protein 173	−2.63	0.002370371

The results of RNA-seq were checked through RT-qPCR and western blot, which were replicated and reported in the related publication in Genes and Diseases Volume 10, Issue 5, September 2023, Pages 1809–1811. The expressions of cGAS, STING, RIG-I, MAVS, NF-kB, interferon and interferon stimulated genes were tested in BT549 cells, MDA-MB-231 cells and in CH12F3 cells, all of which were consistent with RNA-seq results. The details of the confirmation and replications in different cell lines could be searched in the related publication in Genes and Diseases Volume 10, Issue 5, September 2023, Pages 1809–1811.

## 4 Experimental Design, Materials and Methods

### 4.1 Cell culture

Human breast cancer cell lines BT549 were obtained from American Type Culture Collection (ATCC). BT549 cells were cultured in RPMI 1640 medium which containing 10% FBS, and 1% penicillin and streptomycin, 5% CO2, 37 °C.

### 4.2 Reagents

RPMI 1640 medium (SH30809), DMEM medium (SH30022) and Trypsin Protease (SH30042.01) were obtained from Hyclone (UAS). Fetal bovine serum (F-8318) was from Merck (Australia). NU7441 (S2638) was obtained from Selleckchem (USA).

### 4.3 NU7441 Treatment

The BT549 cell line was cultured on cell culture plates, and the DNA-PKcs inhibitor NU7441 was added for 48 h at a concentration of 20 µM (below the IC50 value) when the cell density grew to 80%. Control cells are set up at the same time.

### 4.4 RNA Extraction and Processing

BT549 cells were collected, total RNA was extracted from the cells using Trizol reagent [2], RNA quality was determined using an Agilent 5400 and quantified using a NanoDrop, and the RNA samples were used to construct sequence libraries. The first strand of cDNA was synthesised in M-MuLV reverse transcriptase system using fragmented mRNA as template and random oligonucleotides as primers, followed by degradation of the RNA strand by RNaseH, and synthesis of the second strand of cDNA using dNTPs as raw material under DNA polymerase I. The purified double-stranded cDNA was extracted from the cells using Agilent 5400 and quantified by NanoDrop. The purified double-stranded cDNA was end-repaired, A-tailed and ligated into sequencing junctions, and the cDNA of 370–420 bp was screened with AMPure XP beads, amplified by PCR, and the PCR products were purified again with AMPure XP beads to obtain the final library. After the libraries were constructed, they were initially quantified using a Qubit 2.0 Fluorometer and diluted to 1.5 ng/ul. The insert size of the libraries was then checked using an Agilent 2100 bioanalyzer, and the insert size was determined as expected. After the insertsize met the expectation, qRT-PCR was used to accurately quantify the effective concentration of the libraries (the effective concentration of the libraries was higher than 2 nM) to ensure the quality of libraries. After passing the library inspection, different libraries were pooled according to the effective concentration and the target downstream data volume, and sequenced with Illumina NovaSeq 6000, and 150 bp paired-end reads were generated. Sequenced fragments are converted into sequence data (reads) by CASAVA base recognition of the image data measured by the high-throughput sequencer.

### 4.5 RNA sequencing

RNA-seq transcriptome library was prepared following TruSeqTM RNA sample preparation Kit from Illumina (San Diego, USA) using 5 µg of total RNA. Paired-end RNA-seq sequencing library was sequenced with the Illumina HiSeq xten (2 × 150 bp read length) by Novogene (Beijing, China). The number of RNA-seq data in NCBI is PRJNA872854. R statistical package software EdgeR was utilized for differential expression analysis. KEGG [clusterprofiler(4.10.0)] and GO [clusterprofiler(4.10.0)].

### 4.6 Gene expression data analysis

The raw reads were in FASTQ format. The quality of the reads were assessed using fastqc tool. The adapters, and low quality reads were filtered out from the FASTQ files using fastp tool. TThe fastqc tool was used to re-assess the filtered reads prior to mapping. The FASTQ files after the quality trimming and assessment were used for mapping [3].

The Ensemble Homo sapiens GRCh38 genome was used as reference genome for mapping the clipped reads (https://asia.ensembl.org/Homo_sapiens/Info/Index). Prior to mapping, indexing of reference genome was done using HISAT2 indexing scheme. Subsequently, clean reads were mapped using the HISAT2 tool against the index file [3]. The mapped output files (sam files) were converted into binary files (bam files) using Samtools [4].

The featureCounts tool was used for quantification of mapped reads [5]. Mapped reads were counted at the feature (gene) level with the help of Homo sapiens GRCh38 annotation file (gtf).

Differentially expressed genes were screened using edgeR, and we performed the normalization and base-2 logarithm conversion for the matrix data of each GEO dataset using the limma package in R software. |logFC| >1, *P*-value < 0.05 and adjusted *P*-value < 0.05 were considered to be statistically significant for the DEGs [6]. Furthermore, differentially expressed genes were subjected to gene ID conversion, GO functional annotation and enrichment analysis, and KEGG functional annotation and enrichment analysis using clusterProfiler (v4.10.0) in R studio.

## Limitations

None.

## Ethics Statement

The current work meets the ethical requirements for publication in Data in Brief and does not involve human subjects, animal experiments, or any data collected from social media platforms.

## CRediT Author Statement

**Weifeng Mao:** Data curation, Supervision, Conceptualization, Writing – original draft, Writing – review & editing; **Yanqin Yang:** Supervision, Conceptualization; Writing-review; **Kunyan Li:** Methodology, Data upload, Experimental operations; **Shuailong Zhang:** Data upload, Experimental operations; **Yupeng Gu:** Data upload, Experimental operations; **Jinyan Wang:** Experimental operations.

## Data Availability

Transcriptomic data of BT549 triple negative breast cancer cells treated with 20µM NU7441,a DNA-dependent kinase inhibitor (Original data) (Mendeley Data).
